# Child Migrants in Family Detention in the US: Addressing Fragmented Care

**DOI:** 10.3390/children11080944

**Published:** 2024-08-05

**Authors:** Shela Sridhar, Vasileia Digidiki, Leah Ratner, Dennis Kunichoff, Matthew G. Gartland

**Affiliations:** 1 Division of Global Health Equity, Brigham and Women’s Hospital, Harvard Medical School, 75 Francis Street, Boston, MA 02115, USA; lratner@bwh.harvard.edu; 2 François-Xavier Bagnoud Center for Health and Human Rights, Harvard University, 677 Huntington Ave, Boston, MA 02115, USA; vdigidik@hsph.harvard.edu (V.D.); dkunichoff@hsph.harvard.edu (D.K.); 3 Departments of Internal Medicine and Pediatrics, Massachusetts General Hospital, Harvard Medical School, 125 Nashua St. Suite 725, Boston, MA 02114, USA; mgartland1@mgb.org

**Keywords:** family detention, USA, pediatric, standards of care, immigration detention, pediatric documentation

## Abstract

Background/Objectives: Migrant children in family detention facilities often experience frequent relocations and prolonged stays in precarious living conditions. This frequent relocation results in fragmentation of necessary medical care, leading to delays and inadequate medical care. We aim to highlight the critical need for comprehensive medical documentation in immigration detention facilities, a fragmented health care system and potential harm to these children without appropriate medical documentation. Methods: We conducted a retrospective review of 165 medical records from children detained at the Karnes County Family Residential Center between June 2018 and October 2020 to evaluate the adequacy of pediatric medical documentation in an Immigration and Customs Enforcement (ICE) family detention facility. Specific areas of interest included acute care, nutrition, immunization, developmental screening, and tuberculosis screening. Simple descriptive statistics were used to analyze the data. Results: Only 25% of 418 acute medical care visits included specific diagnoses. There was no documentation regarding follow-up recommendations upon release. 97% of children had a chest X-ray completed for tuberculosis screening, however no follow-up recommendations were documented for those with granulomas. Vaccination histories were inconsistently documented. No nutritional categorizations were completed despite 16% of children being at risk for malnutrition or already malnourished. Conclusions: Our findings revealed significant gaps in documentation, particularly in medical decision-making and clinical reasoning. In a fragmented medical system, inadequate documentation can result in avoidable errors in diagnosis and management. Improving documentation practices is crucial to ensure that all children, regardless of immigration status, receive quality healthcare aligned with national and international standards.

## 1. Introduction

Complete and accurate documentation of a patient’s medical needs and the treatment received is a critical component of pediatric care. Medical records provide important insights into the health of children, serve as the foundation for accurate and timely diagnoses, and are a valuable tool to guide appropriate preventive care. Comprehensive medical records can ensure effective communication between health care providers and patients [1] and become the cornerstone of effective treatments. Documentation that includes insufficient physical examinations, medical history, clinical impressions and, aftercare, severely affects the delivery of appropriate and effective pediatric care. Furthermore, inadequate documentation makes every future interaction with health providers less productive, significantly increasing the chances of harm for children.

Migrant children represent one of the most medically fragile groups of patients with healthcare needs consistently left unmet due to their frequent relocation, unknown medical history [2], and long-term detention in unsuitable living conditions. In these instances, thorough documentation of medical needs is essential to adequate care coordination among multiple healthcare providers and settings, particularly in cases of children with medical complexities. Several studies have documented the unmet health care needs that migrant children have in detention settings and the fragmented care they receive, however, very limited data is available on the quality of documentation in these facilities [3,4,5,6].

For clinicians, understanding what previous diagnoses and interventions have been attempted for individual patients is essential to accurately identifying if conditions are worsening despite adequate treatment, or worsening because of inappropriate treatment. For example, a child with a persistent cough may have a retained foreign body, asthma, or allergies, among other diagnoses. Knowing whether treatment for allergies has been tried and failed is important to rule this out as a potential reason for symptoms and to promptly consider illnesses such as asthma or to ensure that antibiotics and other medications are not given unnecessarily.

Based on data collected from 165 de-identified medical records of migrant children detained in one of the largest US family detention centers between June 2018 and October 2020, this article aims to highlight the paucity of relevant medical information in the medical records in a family detention center. We further aim to describe how this inadequate medical documentation can have longer-term implications for children both in detention and after release. To our knowledge, there is no literature which uses medical records obtained from the facility to describe these challenges in documentation.

### Detention of Children in the US

Despite the international consensus that detention is never in the best interest of children [7,8], more than 70 countries across the world, including the United States continue to deprive migrant children of their liberty, placing them in inhumane and unsuitable detention facilities [9]. The US has reached historic levels of child and family detention in the last decade, driven primarily by the detention program introduced at the end of 2014, the largest since the internment of Japanese Americans in the 1940s [10]. The number of children in US immigration custody has grown tremendously: in Fiscal Year (FY) 2016, more than 50,000 unaccompanied children and more than 75,000 family units were apprehended and placed in detention centers [11] and between 2017 and 2021, 650,000 migrant children were detained by Customs and Border Patrol (CBP) [12].

Furthermore, migrant children are not only detained, but are also forced to endure precarious living conditions in the detention centers for prolonged periods of time, in direct violation of the national minimum standards for the treatment and timely release of detained immigrant children as established by the Flores Settlement Agreement (FSA). The FSA was reached in 1997 in response to the government’s mistreatment of children in immigration detention and set the basic standard for children in US immigration custody, including a mandate that children are detained for the shortest period of time; no longer than 72 h in CBP custody and no longer than 20 days in ICE custody as well as access to sanitation, food, supervision, and medical assistance [13]. However, data has shown that more than one-third of children held, were in ICE custody for more than 72 h between 2017 and 2021 [12]. In 2015, the Flores settlement agreement was expanded to bring child welfare protection to accompanied children in detention centers as well. However, despite the Flores ruling, existing data show that detained migrant children are often held in overcrowded and unsanitary living conditions for extended periods of time [3,6], and receive sub-par medical care [3,4]; evidence of a grave lack of fundamental protections owed to children.

Health care in US Immigration and Customs Enforcement (ICE) facilities is administered by the ICE Health Service Corps (IHSC), which provides care directly to 21 facilities (approximately 100,000 detained people), and oversees the care provided in the remaining 148 non-IHSC-staffed facilities (approximately 169,000 detained people). IHSC is staffed by 1700 health professionals with a budget of $315 million as of FY20 [14]. ICE is expected to operate under the “Performance-Based National Detention Standards” (PBNDS) [15], and the Family Residential Standards (FRS). In 2011, the PBNDS was amended to “improve medical and mental health services”, among other changes [15]. These revisions for medical care, comprising Section 4 of the PBNDS, set several expectations for ICE facilities, including a mandate that facilities have plans to ensure continuity of care for detained individuals both within detention centers and between outpatient providers.

Information regarding a migrant child’s medical history, prior test results, vaccinations, diagnoses, and medications are often missing or scattered across different providers [16]. The child’s journey, the transition to Customs and Border Patrol (CBP) custody, Immigration and Customs Enforcement (ICE), and into the community, creates a very fragmented healthcare experience, which can result in poor handoffs and communication between providers and ultimately in poor overall healthcare for migrant children [2]. As long as children remain in US detention facilities, proper documentation and documentation transfer between facilities and into the community must be practiced and enforced.

Several medical organizations, including the World Health Organization, the American Academy of Pediatrics (AAP) and the American Medical Association (AMA), have described the importance of clinical documentation and communication. The WHO released a policy brief outlining comprehensive standards in pediatric care. Standard #2 of the WHO policy describes the importance of health information systems to ensure “early, appropriate action to improve the care of every child”. This standard includes that every child has a “complete, accurate, standardized, up-to-date medical record, which is accessible throughout their care, on discharge and on follow-up [17]”. Furthermore, every health facility must have a “functional mechanism” for data collection and analysis for quality improvement [17]. These functional mechanisms can include systems like monthly internal audits of medical records, systematic collection of specific information for records to identify differences in medical practices between providers, etc… Similarly, the AMA and AAP have released statements noting that documentation is critical to improving healthcare delivery and patient outcomes [18,19]. These documents should facilitate clinical decision-making, communication, and coordination among clinical care teams, including the patients themselves [19].

Adequate documentation and transparency of medical care reflect the patient’s history and allow for continuity of care in fragmented healthcare systems [20], such as that between detention facilities and community-based care for migrants upon release. However, the existing, limited documentation of ICE’s intake of medical conditions and standards has been described as having delays in screening, denial of medical care and a lack of accountability [21] which does not meet any of the existing national or international standards. This article discusses the importance of complete medical documentation on the quality of care for children in a fragmented health system such as a detention facility.

In December 2021, the Biden administration put a halt to the detention of families in 3 large US family detention centers, the administration has yet to commit to ending family detention altogether. The overarching aim of this study was to provide firsthand evidence on the health status and medical needs of children in immigration detention. Findings of the full study are published elsewhere [22] and highlight the inadequacy of pediatric medical care at a family detention facility.

## 2. Materials and Methods

A retrospective record review was conducted of 165 pediatric medical records from the Karnes County Family Residential Center (KCFRC). The records were obtained through the Refugee and Immigrant Center for Education and Legal Services (RAICES), a legal non-profit organization that provides legal services to children and families and advocates for improved detention conditions.

### 2.1. Sample Size and Setting

Medical records were included for children up to the age of 18 years who passed through the facility between June 2018 and October 2020 and were represented by RAICES. Though a total of 326 medical records were requested by RAICES, ICE released only 165 records, all of which were included in the study. RAICES obtained permission from each child’s guardian for the medical records to be used in immigration-related advocacy and research.

### 2.2. Data Collection and Measures Used

RAICES removed all protected health information identifiers prior to the transfer of records to the research team. The Child Health Immigration Research Team that conducted the study included physicians and advanced practice providers who specialized in pediatrics and family medicine, a medical student, public health faculty with a focus on child protection, and a data analyst. This team collaborated with RAICES to analyze the medical records obtained. Data extraction was completed by the medical clinicians on the research team. The team developed data collection forms based on available and legible information in the medical record. Study data were collected and managed using REDCap electronic data capture tools hosted at Mass General Brigham [23,24]. Specific data forms were developed to identify general demographic information, intake processing data (i.e., past medical history, any follow-up required), nutrition (height and weight), mental health progress record screens, acute medical visits (including vital signs, chief complaint, diagnosis, medications provided and counseling provided), influenza vaccine given (yes/no), dental visit provided during detention (and documentation of any abnormalities), presence of a tuberculosis screen (yes/no and the type of screen completed) and medication dispensed.

### 2.3. Data Analysis

Assessment of nutritional status was made using the WHO height-for-weight z-score classification for acute malnutrition, which is defined as a height-for-weight z-score (standardized measurement) of less than −2 for children under 5 years old and a BMI-for-age z-score of less than −2 for a child 6–18 years old. Children with a z-score between −1 and −2 in height-for-weight or in BMI-for-age were considered at-risk for malnutrition. Stunting was defined as a height-for-age Z-score less than −2 for all children.

Descriptive statistics across all the data collected were calculated to describe the contents of the medical records for the entire cohort. We present categorical data for each variable, describing the results using counts and their respective proportions. We categorized three variables that were collected as numeric data: age (years), days in detention, and nutritional status z-score calculations. We also summarized the specific diagnoses documented in the medical records into general diagnostic categories, as well as summarizing specific countries of origin into regional groups. Depending on the relevance to the measure and post-hoc reviewing of medical records, we either excluded records that had missing values in fields of interest for the descriptive statistic summaries of those fields or we assigned them into a category appropriate after review of their records. Statistical summaries were calculated using R Statistical Software (v4.2.2; R Core Team 2022).

### 2.4. Ethical Review

The ethical review was completed by the Mass General Brigham Institutional Review Board (Protocol Number: 2021P002342) and was exempted from human studies research status given the minimal risk posed by the study to the subjects.

## 3. Results

**Figure 1:**
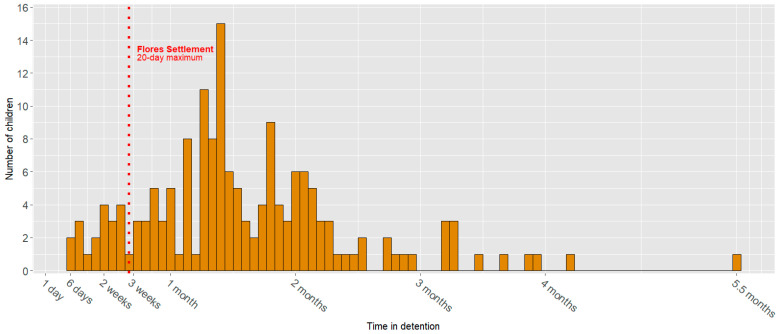
Time in Detention.

A total of 165 records were reviewed of children ranging in age from 6 months to 18 years and originating from 16 different countries. 31 percent of children (50/165) were 5 years old or younger, with 10% of children being 2 years old and younger (16/165). 22 percent (36/165) were between the ages of 6 and 9, and 48% (79/165) were adolescents (age 10–18) (Table 1). Due to the detention practices of ICE during the record collection period relating to the restriction of housing female children with unrelated adult males, 90 percent (148/165) of the study population were male. 88 percent of children (145/165) were detained for more than 20 days at KCFRC, and 8 percent (13/165) were detained for 90 days or more (Figure 1).

A total of 12 languages were documented. Almost 80 percent (132/165) of children were documented as speaking Spanish, and 5 percent of children were documented to speak more than one language. Only 9% of acute medical visits in the records documented the use of interpreters. Of those records that documented acute medical visits, only two referenced the involvement of a Spanish interpreter. Of note, there was no documentation in any of the records regarding the primary language of the provider or agent collecting information. There was no indication as to whether the provider was bilingual or if the guardian was bilingual and interpreting.

### Medical Care

The research team organized medical care into acute medical care and standard pediatric screenings, including tuberculosis screening, influenza, vaccination, and nutrition. Documentation in all areas was evaluated. A total of 418 acute medical visits were recorded across the 136 children (82%, 136/165) in the study population. 56 percent of children (76/136) had more than one acute medical visit documented, with 40 percent of them being seen for more than 3 visits during their detention period. 4 percent (4 children) had more than 8 visits. 18 percent of children (29/165) did not have any medical visits documented.

**Figure 2:**
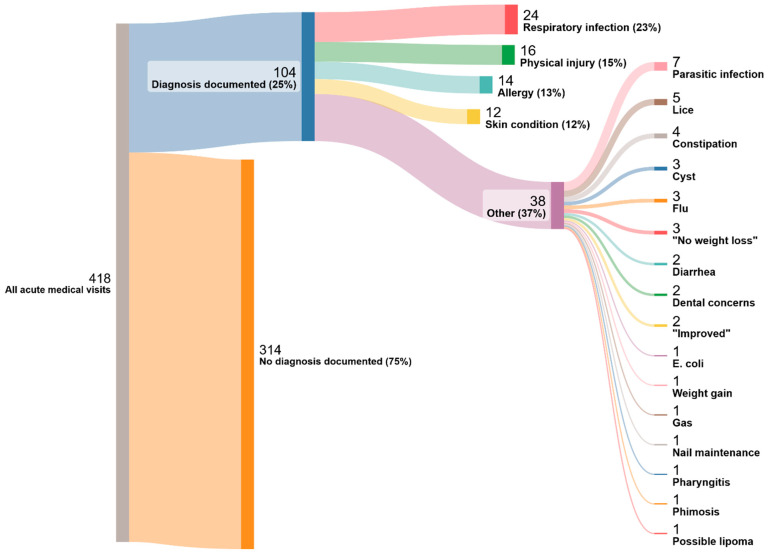
Diagnosis.

Documentation throughout the medical records demonstrated incomplete information regarding both the medical visit and/or recommendations for referral at the time of release when necessary. 25 percent of acute medical visits (104/418) had a diagnosis documented. The most common diagnosis was “respiratory infection” (24/104, 23%). Respiratory infection included the chief complaints: breathing problems, cough, upper respiratory infection, congestion, and Ear, Nose and Throat (Figure 2). TB screening was documented in 97 percent of the records, with 8 (13/155) of children screened for TB being found to have a calcified granuloma on chest X-ray. None of the records documented further evaluation for active tuberculosis (TB). Of the 13 children with a calcified granuloma, two children presented to the acute care facility with a cough, and two children presented with a fever. TB was not evaluated in any of these patients. Three of these children had more than one visit to the acute care facility.

In one medical record reviewed, a sixteen-year-old male from Guatemala presented to the facility with a BMI z-score of −1 who was noted to have a 1 mm calcified granuloma in his right upper lobe, a typical location for TB to be visualized [25]. On the day of arrival he was noted to have a non-productive cough. The plan documented by the provider was to take Cetirizine, increase fluids and cover his cough. There was no documentation that further testing, such as a QuantiFERON Gold or a Tuberculin Skin Test was done for TB, or that this was considered at the time of evaluation [26]. There is no documentation that these records were provided or recommendation to follow up in the community at the time of release. The child was detained for 42 days.

Vaccination history and administration records were handwritten and not legible for the purposes of standard data collection. Only the administration of influenza vaccines by the facility was sufficiently documented. 10 percent of the children (16/165) were missing documentation of influenza vaccine history or administration. Among those with documentation, 32 percent (47/149) of children received at least one influenza vaccine while in detention. Due to the redaction of identifying data, including dates, seasonality was not assessed. No data regarding prior influenza vaccination was included due to the inconsistency of this documentation in the records. Given the inconsistency of vaccination records in the medical record, the sparsity of records, and the inability to read the records that were present, other types of vaccinations were not included in the data collection.

Height and weight were documented at the time of intake for all but 1 child. There was no documentation of Z-score or any alternative classification of children’s nutritional status for children under 5 based on appropriate anthropometric measurements in the records. For children 6–18 years old, a BMI was documented in 89 precent of records (102/115). There was no documentation of the classification of children’s nutritional status based on BMI Z-scores in the records. Z-scores calculated by the research team using height-for-weight and BMI appropriately for age demonstrated that 4 percent (7/162) of children had moderate or severe acute malnutrition (Table 2). An additional 12 percent (19/162) were at risk for malnutrition. 23 percent of children (37/163) had a height-for-age Z-score of less than −2, indicating the presence of moderate or severe stunting (Table 2).

The medical records did not contain any documentation for follow-up recommendations outside the facility upon release or repatriation of children to their country of origin. There was no documentation of whether families were provided guidance or instructions to seek further medical care for identified health conditions. In one instance, a 16-month-old child from Brazil who had multiple visits to the facility’s urgent care clinic for cough, congestion, and difficulty breathing with a known history of albuterol use. Her symptoms included recurrent and nighttime cough, which is a classic symptom of reactive airway disease or asthma in younger children. There was no documentation of steroid administration or long-acting controller medications, which suggests incomplete medical care for asthma [27]. Furthermore, there was also no documentation or indication of any type of referral to a specialist or a recommendation to follow up with a primary care physician for asthma control was discussed or considered.

## 4. Discussion

The results demonstrate inadequate documentation in key areas of the medical record for children in ICE family detention centers, including interpreter utilization, acute medical care, TB screening, influenza vaccine administration, and nutrition. This represents a potential harm in circumstances where children return for follow-up or are referred for a higher level of care, as subsequent providers will not have access to previous testing and decision-making processes leading up to the visit [28]. While inadequate documentation can lead to frustration on the part of subsequent providers, it is particularly important to address as it can lead to errors in patient care, such as sentinel clinical events (including medication interactions, incorrect treatments resulting in serious side effects, etc). Complete documentation is essential for the quality of care in a fragmented system such as a detention facility where children are held for limited periods of time and are then released to the community in which a new health system is responsible for providing adequate medical care. As noted, diagnosis and documentation of clinical reasoning to reach a conclusion is critical in any medical record. However, only 25% of acute medical visits had a diagnosis documented, and none of the records reviewed had a description of the provider’s clinical reasoning or alternative diagnoses considered. The inadequacy of documentation around interpretation and communications raises questions regarding the conditions under which the medical history was received, and the medical care was provided.

Further, the study showed that 8 percent of children had calcified granulomas on their chest X-rays with 4 of those 13 children (23%) reporting symptoms of cough and fever, which may indicate the existence of tuberculosis. This is of particular concern in this population, which is at much higher risk based on the endemicity of TB in their home countries. Though calcifications can be benign, depending on the size of the calcified nodule in children, they can also be indicative of alternative diagnosis, including malignancy, tuberculosis, and other infectious conditions such as aspergillosis or Histoplasma which all require prompt attention [29]. Therefore, children with calcifications found on an X-ray must have close follow up with a pediatric-specific provider. Furthermore, for calcifications found to be tuberculosis, children are at higher risk of conversion to active TB than adults [30] which has the potential to spread within communities and create a public health emergency. This recommendation for follow-up was not documented in any of the records reviewed. Complete data and follow-up recommendations regarding infectious diseases are particularly critical given the risk of spread and public health implications in detention settings and in communities when children are released.

In addition to infection risk, key data regarding growth and development were missing. This information is critical for long-term outcomes when children are detained for several days. Almost 40 percent of the study population had some form of undernutrition or were at-risk of malnutrition at the time of intake into KCFRC. Although there was no systematic documentation of nutritional assessment based on anthropometric measurements at intake, several children were documented to receive nutritional supplementation during acute illnesses. Even in these instances, children were not given a diagnosis of weight loss or malnutrition and there was no systematic approach to monitoring and treatment. Undernutrition has significant long-term consequences and early intervention is critical. The first 1000 days of life is critical to reversing the impacts of malnutrition [31]. Identifying the children and intervening at each point of contact with the medical system is essential to preventing long-term impacts on the health and development of children, including susceptibility to infections [32], cognitive and motor delays, and decreased productivity in adulthood [33]. However, the classification of children with malnutrition was not noted in any of the records reviewed at the time of intake. Furthermore, there was no follow-up recommended or description of urgency for follow-up at the time of release.

Handoffs within the medical systems should document the expectations regarding the patients’ condition, testing that is still outstanding and should be followed up on, ongoing care and any unresolved issues [20]. However, as this study illustrated, there was no documentation of further testing or follow up being recommended after release. This is especially concerning in the case of infectious disease processes such as TB testing and vaccine-preventable diseases which have the potential to create public health emergencies both within detention, where there is a high risk of spread [34,35], as well as within the community after release. The vaccination records were often not present and when they were present, they were generally illegible. Furthermore, there was no evidence that families were released with medical records, which is consistent with previous studies in which clinicians in the community describe a lack of continuity and inability to access records during detention [36].

While neither the PBNDS nor the FRS clearly delineates what the quality of documentation should be and what must be documented in regard to medical care, the PBNDS does indicate that complete health records should be kept in accordance with accrediting body standards. Per these standards, at a minimum, documentation of a clinical record should include four key components, the: Subjective, Objective, Assessment and Plan (SOAP); a standard template used among clinicians. In the subjective section, a comprehensive clinical history, including duration of symptoms, associated symptoms and relevant family, social (including language barriers and need for an interpreter) and medication history should be included. The objective section should include all relevant physical exam findings including vital signs. The assessment section should describe the thought process of the provider, the alternative diagnosis that were considered and the different aspects of the clinical case which point the provider to a specific diagnosis, as well as a final likely diagnosis [37]. The plan should be a succinct description of what interventions are recommended and any follow up for the condition. When these key components of a clinical visit are missing, it can mean that particular diagnoses were not considered and relevant questions were missed or that the appropriate treatment plan was not executed, which can result in patient harm. It may also mean that appropriate care was provided, but not documented. However, in both instances, if the child is released and continues to suffer from a particular condition such as a cough or weight loss, providers must be able to understand what medical care has been provided. Incorrect and incomplete data is not acceptable in other US-based clinical settings, and should not be tolerated in US-based detention facilities.

The existing standards also indicate that documentation of medical care must exist and that at the time of medical transfer, completed medical records must be sent to the accepting facility and that informed consent must be documented for care [15,38]. The documents also indicate the need to identify the completion and results of screening tools such as tuberculosis, intake medical history and administration of medication. This structured approach, in which all components of the clinical history are included, is associated with higher quality of documentation and ultimately information exchange when patients, and in this case, children, are referred to other facilities [39]. However, despite the presence of these governing documents, ICE is not legally bound by these standards and thus does not have consequences for non-adherence, resulting in varying reliability of compliance to these standards, depending on the facility [40]. Additionally, the vague wording of these standards further drives inadequate accountability and enforceability of the standards outlined. Therefore, it is the needs of for-profit organizations, such as CoreCivic and GeoGroup, that guide the standards of medical documentation and quality of care, rather than patient needs [41]. To protect the basic rights of children, ICE must be held to the most basic standards of pediatric medical care, which includes the need for adequate documentation and family access to their medical records at the time of release.

Adequate documentation is critical to ensuring both quality of care and adherence to clinical standards. Oversight mechanisms must be in place to ensure that basic clinical standards are being met. There are currently no systematic reviews in place to monitor health outcomes with the exception of death reviews [5]. A report by the Department of Homeland Security Office of Inspector General noted that when oversight inspections do occur, they are too infrequent and are insufficient to “examine actual conditions or identify all compliance deficiencies [5]”. This lack of oversight is deadly to children. Several news reports have described preventable pediatric deaths [42,43,44]. This cannot be the standard by which medical care in any context is accepted. If children remain in any type of detention facility, pediatric illness must be addressed well before serious morbidity and mortality. Conditions such as asthma and dehydration are easily treatable and severe attacks are preventable with the proper care. Government agencies and the private companies that they employ must adhere to standard clinical guidelines based on US standards of care if they are to provide medical care for children in these facilities

According to the Office of the Inspector General (OIG) 2021 report, there are many challenges to implementing the model of care that is delineated in PBNDS. These include vacancies in medical staffing positions due to the remote location of many facilities, lack of incentives for employees, and limited resources for recruitment [45]. Additionally, the report notes that while private contractors who do not uphold staffing requirements or timeliness of care can be sanctioned by withholding funds and moving detainees out of the facility, these penalties have limited efficacy in initiating changes as contracts may not be specific enough and contractors may have sufficient demand outside of ICE to switch entities for beds [43]. Further challenges include access to language-appropriate care: a 2016 DHS report notes that medical and mental health providers are often not bilingual, and materials about rights and procedures are often only provided in English. Investigations are unclear as to whether centers appropriately use interpreter services [46]. In this study, there was virtually no documentation of the patient’s preferred language or proficiency in English and only 9 percent of acute visits had interpreters documented. It is unclear whether this was due to the lack of need for interpreters, the lack of availability, or provider neglect.

Allowing these issues to continue prevents children from getting the medical care that is their human right. Many immigration detention facilities are run by private corporations such as GeoGroup and Core Civic, including KCFRC which was run by GeoGroup at the time of the study. Privatization placed the emphasis of medical care on profit-seeking behavior rather than adequate medical care. This emphasis results in understaffing, delayed transfers for medical care deterring families from seeking medical care while in detention [47]. These challenges can be overcoming by shifting the emphasis towards providing quality, timely medical care to children, rather than profit.

### Limitations

Limitations of our study include challenges to generalizability. The study sample is confined to a 27-month period at one family detention facility and represents only half of the medical records were initially requested from ICE (165/326). This, combined with the fact that 90 percent of the study cohort are male, prevents the research team from making broader generalizations about the healthcare provision to the entire population of migrant children detained in the US and instead confines the authors to comment only on what has been documented. Within that, however, there is substantial missing documentation for several critically important fields of medical information, including, but not limited to, demographics, nutrition, and vaccinations. For example, 10 children’s medical records contained no information about screening for tuberculous and 16 children’s medical records contained no information on influenza vaccines. Furthermore, we do not have documentation from outside of the facility, if any, included in the records, making it impossible to assert whether care outside the facility was ever offered or if vaccinations such as flu were received prior to entry. As a result, we are also unable to provide information regarding comprehensive care that may have been provided to children. Finally, the records do not provide an account of children and families’ experience of health medical care, while in detention, and may not capture inadequate communication around medical care or attempts by families to seek care.

Despite these limitations, to the best of our knowledge, this study is the first comprehensive review of pediatric-specific medical records from an immigration detention facility and shines a light on the inadequate medical care and oversight in detention facilities. Our study has many strengths, including a systematic and rigorous approach to identifying missing data and the contextualization of data in practical and real-world medical practices. By documenting and highlighting the gaps in medical documentation, we provide the first steps to ensure that a robust oversight and quality improvement mechanism within immigration detention facilities is achieved.

## 5. Conclusions

Adequate documentation, particularly in fragmented systems, is critical in providing appropriate medical care to children. Our study sheds light on the inadequate documentation which can exist in immigration detention centers. All children, regardless of immigration status, are entitled to quality medical care as described by both US and international law, and the necessary oversight must be in place to ensure that agencies are held accountable to these standards. The lack or absence of these safeguards, directly impedes the ability to provide rights-based pediatric care. In order to ensure that facilities are adhering to basic clinical standards of care, as outlined by US medical organizations, adequate medical documentation must be present. Without sufficient oversight and the necessary information to inform inspections and reviews, we place the community, within detention facilities and those where children are released, at risk of infectious spread and the children themselves at risk of severe acute and long-term complications and even death.

In addition to ensuring that documentation is present to promote oversight and accountability, medical documentation must be improved to develop internal quality improvement mechanisms to improve patient outcomes. Systematic reviews of medical records can be a tremendous source of information for providers within systems to highlight areas of success, as well as where systems can be strengthened. These documents serve as roadmaps of care for providers outside of detention facilities who will later care for these children. In order to truly translate the ideal of healthcare as a right for all children, there must be a thorough assessment and implementation of standardized clinical documentation in line with national standards in medical facilities located in immigration detention centers. All children deserve our advocacy to ensure they receive rights-based quality care.

## Figures and Tables

**Table 1:** Sample Demographics.

Age at Arrival Grouping (Years)	N (%)
<2	16/165 (9.7%)
2–5	34/165 (20.6%)
6–9	36/165 (21.8%)
10–17	79/165 (47.9%)
**Gender**	
Female	15/163 (9.2%)
Male	148/163 (90.8%)
**Region**	
Africa	4/163 (2.5%)
Asia	1/163 (0.6%)
Europe	3/163 (1.8%)
Northern Triangle (El Salvador, Guatemala, Honduras)	92/163 (56.4%)
Remainder Central America/Carribean	43/163 (26.4%)
South America	20/163 (12.3%)
**Languages spoken**	
Spanish	122/165 (73.9%)
Creole	20/165 (12.1%)
Indigenous Lang. of Guatemala	7/165 (4.2%)
Portuguese	7/165 (4.2%)
French/Lingala	2/165 (1.2%)
Romanian	2/165 (1.2%)
Mandarin	1/165 (0.6%)
Russian	1/165 (0.6%)
Not specified	3/165 (1.8%)
**Days in detention**	
0–20 days	20/164 (12.2%)
21–89 days	132/164 (80.5%)
+90 days	13/164 (7.9%)

**Table 2:** Rates of Malnutrition.

Nutritional Condition	Categories	Z-Score Ranges	Full Sample (N = 165)	0–4 Years Old (N = 42)	5–18 Years Old (N = 123)
Malnourishment (WFH for 0–4 yo, BFA for 5+ yo)			N (%)	N (%)	N (%)
	Any level of malnourishment	≤−2	7/163 (4.3%)	3/42 (7.1%)	4/121 (3.3%)
	Severe	≤−3	0/7 (0%)	0/3 (0%)	0/4 (0%)
	Moderate	≤−2 & ≥−3	7/7 (100%)	3/3 (100%)	4/4 (100%)
	No malnourishment	>−2	156/163 (95.7%)	39/42 (92.9%)	117/121 (96.7%)
	At risk	≤−1 & >−2	19/156 (12.2%)	7/39 (17.9%)	12/117 (10.3%)
Stunting (HFA)					
	Any level of stunting	≤−2	37/163 (22.7%)	5/42 (11.9%)	32/121 (26.4%)
	Severe	≤−3	9/37 (24.3%)	0/5 (0%)	23/32 (71.9%)
	Moderate	≤−2 & ≥−3	28/37 (7.6%)	5/5 (100%)	9/32 (28.1%)
	No stunting	>−2	126/163 (77.3%)	37/42 (88.1%)	89/121 (73.6%)
	At risk	≤−1 & >−2	35/126 (27.8%)	6/37 (16.2%)	29/89 (32.6%)

## Data Availability

Data available on request due to ethical restrictions. The data presented in this study are not publicly available due to restrictions from the 3rd party and concern for client privacy and protections.
